# A parallel heterogeneous policy deep reinforcement learning algorithm for bipedal walking motion design

**DOI:** 10.3389/fnbot.2023.1205775

**Published:** 2023-08-08

**Authors:** Chunguang Li, Mengru Li, Chongben Tao

**Affiliations:** ^1^School of Computer and Information Engineering, Changzhou Institute of Technology, Changzhou, Jiangsu, China; ^2^School of Electronic and Information Engineering, Suzhou University of Science and Technology, Suzhou, China

**Keywords:** gait optimization, biped robot, Deep Deterministic Policy Gradient, experience replay, parallel heterogeneous strategy

## Abstract

Considering the dynamics and non-linear characteristics of biped robots, gait optimization is an extremely challenging task. To tackle this issue, a parallel heterogeneous policy Deep Reinforcement Learning (DRL) algorithm for gait optimization is proposed. Firstly, the Deep Deterministic Policy Gradient (DDPG) algorithm is used as the main architecture to run multiple biped robots in parallel to interact with the environment. And the network is shared to improve the training efficiency. Furthermore, heterogeneous experience replay is employed instead of the traditional experience replay mechanism to optimize the utilization of experience. Secondly, according to the walking characteristics of biped robots, a biped robot periodic gait is designed with reference to sinusoidal curves. The periodic gait takes into account the effects of foot lift height, walking period, foot lift speed and ground contact force of the biped robot. Finally, different environments and different biped robot models pose challenges for different optimization algorithms. Thus, a unified gait optimization framework for biped robots based on the RoboCup3D platform is established. Comparative experiments were conducted using the unified gait optimization framework, and the experimental results show that the method outlined in this paper can make the biped robot walk faster and more stably.

## 1. Introduction

As robotics research continues to advance, robots have development trends in various fields (Singh et al., 2022). Particularly, the biped robot, as a kind of bionic robot, has higher adaptability, operation ability and interactivity due to its human-like double-leg structure. However, gait optimization, as a key issue in the stable walking technology of biped robots, has always been a hot and difficult point in the field of robotics research. In the early stage of gait optimization research, traditional control methods were widely used in the field of gait control of biped robots. The main categories include the following: fixed gait control methods, such as human walking parameters (Horn et al., 2020; Rosa and Lynch, 2022), zero moment point (ZMP) (Farid et al., 2022; He and Yamamoto, 2022), etc. Paredes et al. proposed an approach based on a linear inverted pendulum model to deal with biped gait, but the auxiliary input of gait parameters is required to achieve stable walking of biped robot (Paredes and Hereid, 2022). Despite their low computational requirements, the aforementioned methods exhibit limited flexibility, as they are tailored to a single scenario and are susceptible to external perturbations. Feedback-based gait control methods, which refer to real-time adjustments based on the external environment and internal state perceived by the robot, such as passive walking (Safartoobi et al., 2021), fuzzy logic control (Maroger et al., 2021; Dong et al., 2022), etc. These methods also have some drawbacks, with high requirements for sensors and computing power. Optimization-based gait control methods, which mainly refer to the theory of optimization algorithms (Duburcq et al., 2020), inevitably require not only a large number of calculations, but also complex control planning. Currently, Genetic Algorithm (GA) and Particle Swarm Optimization (PSO) have been applied to optimize the gait of biped robots (Tao et al., 2021; Liu et al., 2022a). Kashyap et al. demonstrated the superiority of PSO over GA in gait optimization by their study (Kashyap and Parhi, 2021). However, since the gait control of the biped robot is a multi-objective optimization problem, too many parameters need to be set for the biped robot, which may cause the PSO algorithm to fail to obtain the optimal value during optimization, resulting in the limitation of the walking speed of the robot. Generally speaking, most traditional research methods have problems such as low efficiency, poor robustness, and low generalization.

The advent and progression of reinforcement learning (RL) has opened up new avenues for gait control of biped robots (Kasaei et al., 2021). RL methods do not depend on the level of robot hardware and also allow parameter debugging in a short time (Castillo et al., 2020). However, the extraction of biped robot state features based on reinforcement learning approach is not an easy task (Wu et al., 2020). Combined with more stable machine learning networks, RL has gradually developed into DRL, which provides a new solution for dealing with high-dimensional features (Niroui et al., 2019; Clegg et al., 2020). DRL can better handle complex state spaces from real-world scenarios of biped robots. DRL is able to automatically learn abstract, high-level feature representations and acquire strategies for control from environmental feedback through continuous training. Such an approach to learning strategies is more stable and can effectively avoid the effects of inaccurate dynamic models (Rodriguez and Behnke, 2021). DDPG is a DRL algorithm for continuous control, which extends the application of RL by learning end-to-end to control directly based on the original inputs and outputs (Li et al., 2019). DDPG-based biped robot walking revolves around multiple action strategies based on Markov Decision Process (MDP) to learn algorithms (Tao et al., 2022). Compared with the greedy policy in parallel algorithm training in Tao et al. (2022), the algorithm proposed in this paper is better at dealing with the instability of parallel architecture. Although the biped gait control method based on DDPG enables the algorithm to converge faster through the experience replay mechanism, the same problem exists. The excellent performance of the experience replay mechanism requires many conditions, such as larger storage space, more training samples and more advanced hardware. Moreover, the current experience replay methods do not make full use of good experience. Additionally, the biped gait control method based on DDPG also lacks effective reward functions.

Building upon the aforementioned discourse, a parallel heterogeneous DRL algorithm to optimize the walking gait of biped robots is proposed by considering the algorithmic efficiency, experience utilization, and insufficient reward of DRL. Specifically, the main contributions of this paper are summarized in the following aspects.

1) The designed algorithm is based on the DDPG algorithm as the main architecture, which uses the multi-threading function of CPU to run the intelligent body in parallel to interact with the environment. The network model and learning experience are shared through a multi-threaded approach, allowing a large number of experience samples to be acquired at a low cost. In addition, experience samples filtered and stored by heterogeneous experience playback mechanism are used to sample excellent and common experience samples with different sampling probabilities to speed up training.
2) The gait of a biped robot is characterized by periodicity. In this paper, a sinusoidal periodic curve is used to construct the foot trajectory reference of the biped robot when walking. The reference has a positive effect on constructing positive reward incentives to generate good experience samples, which ultimately improves training efficiency. The foot velocity and ground reaction force during the cycle are also taken into account to provide assurance for the stability of the robot when walking.
3) Different environments and different biped robot models pose challenges for different optimization algorithms. Based on the RoboCup3D platform, a unified biped robot gait optimization training framework is constructed. The solution defines standard gait optimization tasks and provides a common environment interface for various optimization algorithms. When comparing the effects of different algorithms on the gait of the biped robot, only the algorithm module needs to be replaced, which reduces the baseline error caused by the difference in the environment.

The rest of the paper is organized as follows: Section 2 presents the work related to gait optimization of biped robots. Basic definitions and citations needed for this paper are given in Section 3. Section 4 presents the proposed algorithm, and Section 5 gives a general bipedal robot gait optimization training framework. In Section 6, the robustness of proposed method is verified by comparative experiments. In the end, the research is concluded in Section 7.

## 2. Related work

The traditional gait optimization method first plans the trajectory of each joint in the gait of biped robot according to the expected walking requirements, and then calculates the trajectory of each joint angle by using an inverse kinematic model. Generally speaking, gait optimization is to keep the biped robot stable and avoid falling down when walking, as well as to make the walking posture look beautiful. Due to complexity of the multi-link structure of biped robot, many researchers have obtained various simplified models through reasonable simplifications. The 3D Center of Gravity ZMP (COG-ZMP) model proposed by He et al. considered the effect of center of gravity acceleration on the stability of robot motion and used centroidal viscoelasticity control to stabilize the whole-body posture during the landing phase (He and Yamamoto, 2022). Safartoobi et al. modeled the impulsive hybrid dynamics based on classical bipedal passive walking dynamics model (Safartoobi et al., 2021). Elhosseini et al. used an improved Whale Optimization Algorithm to optimize parameters of biped robot, and results demonstrated that the method was with better convergence characteristics and smaller error (Elhosseini et al., 2019). Tao et al. used a parallel multigroup PSO to optimize key parameters in the gait model, and results showed that the method were stable during the switching between single-support phase and dual-support phase (Tao et al., 2021).

Since RL does not require an accurate mathematical model, many researchers have applied RL methods to control biped robots. Castillo et al. used a RL mechanism that combined physical insights gained from the hybrid nature of walking dynamics and the recognized zero dynamics approach for three-dimensional biped walking, which eventually produced stable limit walking cycles (Castillo et al., 2020). Liu et al. combined Q-learning with radial basis function network to solve the walking problem of biped robot in different terrain (Liu et al., 2018). RL and deep learning have been combined by researchers to propose the DRL approach. The most famous algorithm for DRL is Deep Q-learning (DQN) (Liu et al., 2022b). Wu used passive dynamic walker as a reference and trained the controller using DQN algorithm to make the robot possible to walk on different slopes (Wu et al., 2019). Melo et al. used Proximal Policy Optimization to train gait of the humanoid robot where the output was the step size and the direction of the robot (Melo et al., 2021). Chun et al. proposed a deep reinforcement learning algorithm for gait optimization of biped robot based on realistic environmental. In their study, DDPG was used as a parameter setting method for a biped robot on a real-world environmental testbed, where the trained robot could maintain the walking stability even with short walking times and long strides (Chun et al., 2023).

## 3. Preliminaries

In the interaction between RL and environment, agents learn appropriate actions to maximize the reward signal or achieve a certain goal. RL can be formalized as MDP. An MDP is defined as a four tuple (*S, A, P*_*a*_, *R*_*a*_), where *S* represents a finite set of states, and *A* represents a finite set of actions. $P_{a}(s,s^{\prime })=\mathrm{Pr}(s_{t+1}=s^{\prime }|s_{t}=s,a_{t}=\text{a}$ indicates the probability of transitioning to state *s*′ from *s* under the time *t* taking action of *a* and $R_{a}\left(s,s^{\prime }\right)$ indicates the current reward.

The objective of RL is in order to find a mapping function π (usually called “strategy”) from state to action which maximizes the cumulative rewards. The cumulative rewards can be defined as follows


(1) $$\begin{array}{l} R_{t}=\overset{T}{\underset{i=t}{\sum }}\gamma^{i-t}r\left(s_{i},a_{i}\right), \end{array}$$

where *r*(*s*_*i*_, *a*_*i*_) is the reward obtained by taking action *a*_*i*_ in the state *s*_*i*_; γ represents the attenuation factor and γ∈[0, 1].

In the process of optimization, the expected value of *R*_1_ from a start state distribution is defined as


(2) $$\begin{array}{l} J=E_{r_{i},s_{i}\sim E,a_{i}\sim \pi }\left[R_{1}\right]. \end{array}$$

Next, the action value function *Q* is defined. The expectation of the obtained feedback value is *Q* after taking action *a*_*t*_ according to strategy π, and the calculation process of *Q* is given as


(3) $$\begin{array}{l} Q^{\pi }\left(s_{t},a_{t}\right)=E_{r_{i\geq t},s_{i>t}\sim E,a_{i>t}\sim \pi }\left[R_{t}|s_{t},a_{t}\right]. \end{array}$$

Furthermore, the Bellman Equation can be defined as Lillicrap et al. (2015)


(4) $$\begin{array}{ll} Q^{\pi }(s_{t},a_{t})= & E_{r_{i\geq t},s_{i>t}\sim E}[r(s_{t},a_{t})\\ & +\gamma E_{a_{t+1}\sim \pi }[Q^{\pi }(s_{t+1},\pi_{s_{t+1}}]]. \end{array}$$

DRL is a variant of RL that uses deep learning techniques to improve learning efficiency and accuracy. DDPG is a representative DRL method that is effective in solving the continuous control problem by generating Q values or action probabilities through a deep neural network and outputting continuous actions. DDPG uses two types of networks: actor networks and critic networks, which continue the idea of DQN by using a fixed target network. Each network includes both a target network and an estimation network. The traditional Policy Gradient method uses a random strategy to acquire actions by sampling the distribution of the current optimal strategy. In contrast, DDPG adopts a deterministic strategy, with the actor network taking the current state as input and outputting a deterministic action. The critic network is used to fit the state-action value function, with its input consisting of the current state and actions generated by the actor network, and the output being the current state-action pair Q value. This Q value is used to update the parameters of the actor network.

## 4. Methodology

### 4.1. Parallel heterogeneous policy DRL algorithm

**Figure 1:**
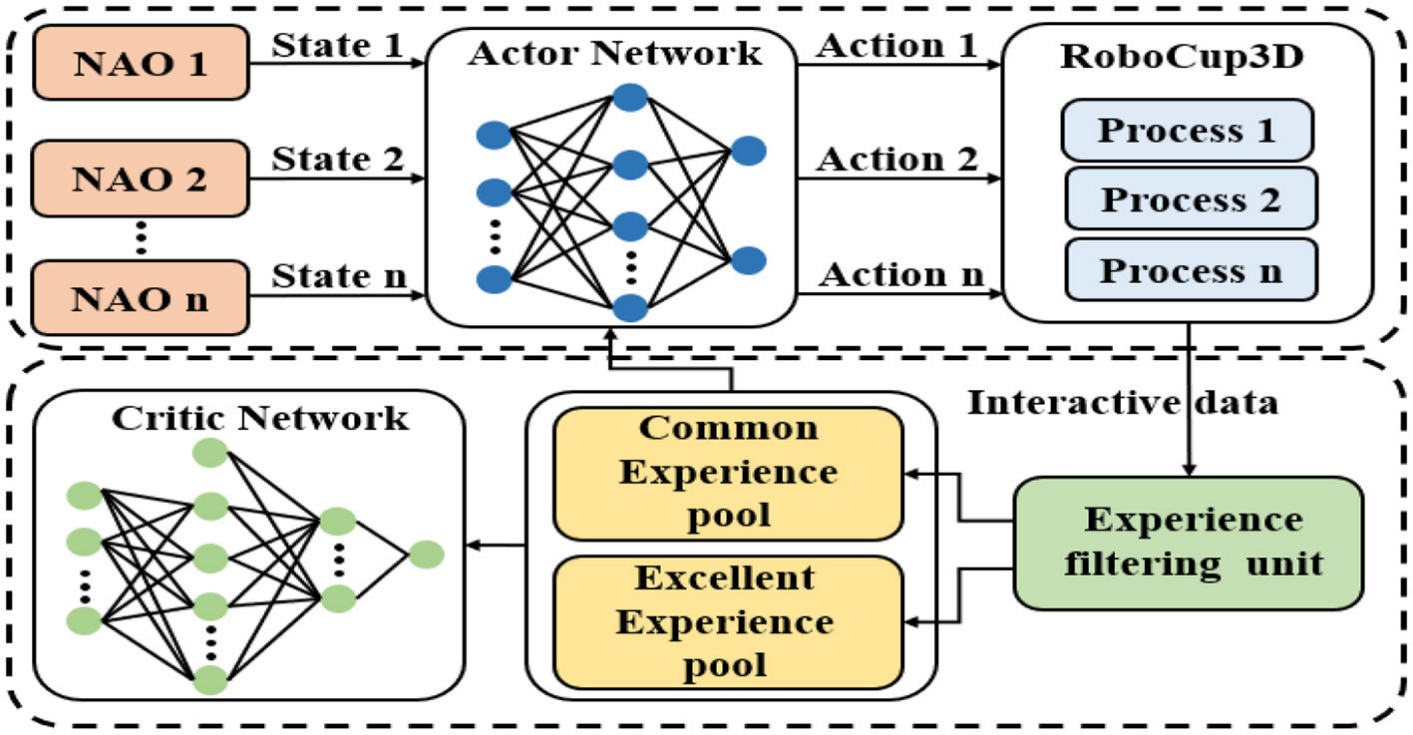
The process of gait optimization with Parallel DDPG. Multiple DDPG agents execute in parallel through the multi-thread function, and achieve global goals by sharing empirical data and strategies.

The framework of the proposed method is shown in Figure 1. In the context of this manuscript, multiple agents are executed in parallel by utilizing the multithreading capabilities of computer CPUs to achieve global goals by sharing empirical data and strategies. Notably, the DDPG algorithm serves as a pivotal component of the framework outlined in this paper. Therefore, the proposed method in this paper is also known as the parallel heterogeneous policy DDPG algorithm (Parallel DDPG).

DDPG is a method of differentiating strategy. The behavior strategy is random but the evaluation strategy is deterministic (Wang et al., 2016). Random strategy can explore and produce a variety of behavioral data, which can be used by deterministic strategy for improvement of the strategy. DDPG is an Actor-Critic algorithm based on Deterministic Policy Gradient (DPG). The learning process of *Q*(*s, a*) in critics is the same as that in Q-learning.


(5) $$\begin{array}{l} \frac{\partial L(\theta^{Q})}{\partial \theta^{Q}}=E_{S,a,r,s^{\prime }\sim D}[(\mathrm{Target}Q\\ \;-Q(s,a|\theta^{Q})\frac{\partial Q(s,a|\theta^{Q})}{\partial \theta^{Q}})]. \end{array}$$

(6) $$\begin{array}{l} \mathrm{Target}Q=r+\gamma Q^{\prime }\left(s^{\prime },\pi \left(s^{\prime }|\theta^{\mu^{\prime }}\right)|\theta^{Q^{\prime }}\right). \end{array}$$

The value gradient on the actor network is


(7) $$\begin{array}{l} \frac{\partial J\left(\theta^{\mu }\right)}{\partial \theta^{\mu }}=E_{S}\left[\frac{\partial Q\left(s,a|\theta^{Q}\right)}{\partial a}\frac{\partial \pi \left(s|\theta^{\mu }\right)}{\partial \theta^{\mu }}\right]. \end{array}$$

(8) $$\begin{array}{l} \nabla_{\theta }J_{\beta }\left(\mu_{\theta }\right)={\int_{S}\rho^{\beta }(s)\nabla_{\theta }\mu_{\theta }(s)Q^{\mu }(s,a)|}_{a=\mu_{\theta }}ds\\ \;=E_{s\sim \rho^{\beta }}\left[{\nabla_{\theta }\mu_{\theta }(s)Q^{\mu }(s,a)|}_{a=\mu_{\theta }}\right]. \end{array}$$

MSE (Mean Squared Error) is adopted as the loss function, and Monte Carlo method is used to obtain unbiased estimation when calculating the gradient expectation of strategy. The gradient on the critic and actor network can be used to update network. Due to the reference to the structure of DQN, there is an additional target network. Although both DDPG and DQN use target networks to improve convergence and stability, parameters are updated in different ways. DDPG proposes a soft update instead of copying parameters directly to the target network. Specifically, DDPG sets up a copy for actor network and a copy for critic network separately as their own objective network. The updating formula for weight of target network is defined as


(9) $$\begin{array}{l} \theta^{\prime }\leftarrow \tau \theta +\left(1-\tau \right)\theta^{\prime }, \end{array}$$

where τ < < 1.

The change of target value calculated by the target network will be very small which improves the stability of learning to a certain extent. In the environment-exploring phase of algorithm, DDPG adds a random noise sampled from a noise process *N* to the strategy performer, so as to take both exploration and exploitation into account, as shown in Equation (10) (Lillicrap et al., 2015).


(10) $$\begin{array}{l} \mu^{\prime }\left(s_{t}\right)=\mu \left(s_{t}|\theta_{t}^{\mu }\right)+N. \end{array}$$

DDPG adopts the experience replay mechanism which is similar in DQN. In the learning phase, a buffer with limited size is set up to store the tuples of state transition in the training process, i.e., (*s*_*t*_, *a*_*t*_, *r*_*t*_, *s*_*t*+1_). When the buffer is full, the old tuple is discarded. In the process of training, these tuples of state transition are randomly sampled from the buffer to update the critic network and the actor network, which enables the algorithm to learn from some irrelevant state transitions.

The proposed Parallel DDPG algorithm greatly improves the diversity of experience samples through the parallel training of multiple biped robots controlled by multiple threads. But each biped still uses the same actor network and critic network. All robots feed their state data back to the environment process according to the same actor network. Actor network and environmental processes interact in parallel to collect experience samples.

It is worth noting that the benefits of the parallel framework were previously demonstrated in our work (Tao et al., 2022). However, in contrast to our previous approach of selecting the corresponding at value based on the maximum *Q*_*i*_ value, the parallel DDPG algorithm proposed in this paper collects the control outputs of each DDPG agent and performs a simple voting process, taking the mode as the final control output, ie, $a=\mu \left(s|\theta^{\mu_{j}}\right)$, $Q\left(s,a_{j}|\theta^{Q_{j}}\right)=\text{median}\left(Q\left(s,a_{i}|\theta^{Q_{i}}\right)\right).$ This approach improves the robustness of the algorithm. Our framework setting is particularly effective in addressing the issue of instability in parallel models. By integrating the outputs of multiple models, we are able to smooth the results and reduce fluctuations, which is a crucial improvement for the parallel framework. The instability problem is particularly prominent in parallel architectures, and our approach provides a promising solution to this challenge.

The experience generated by the interaction is stored in the experience filtering unit, and the actual experience playback is performed using two experience pools in this work. The two experience pools are the common experience pool and the excellent experience pool. The common experience pool is similar to the experience pool of the traditional DDPG algorithm. The excellent experience pool is used to store excellent experience with high rewards. Experience stats are added each round. At the end of each round, the stored experience is filtered by the experience filtering unit and sorted into the corresponding experience pool. The size of common experience pool and excellent experience pool is fixed. The probability of sampling from the Excellent experience pool and updating the parameters is α and the probability of sampling from the common experience pool and updating the parameters is 1−α. Parallel DDPG with the heterogeneous experience replay mechanism is shown in Algorithm 1.

**Algorithm 1:**
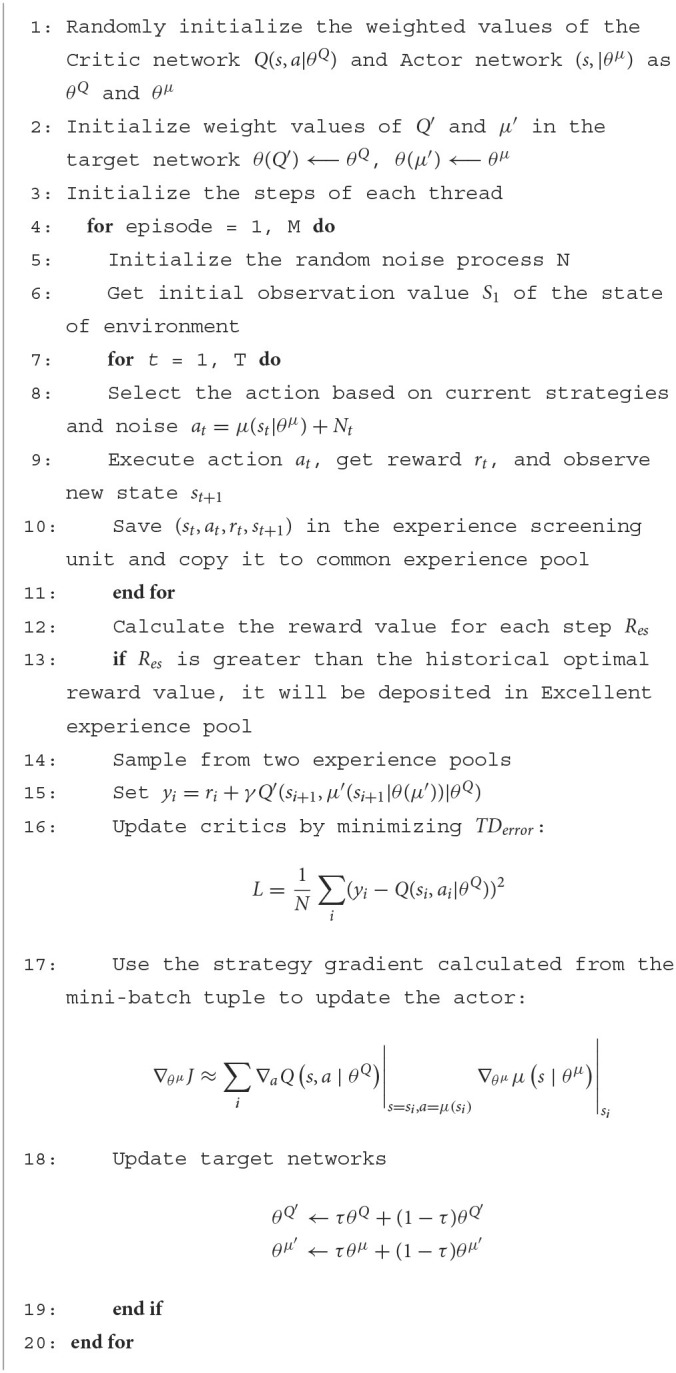
Parallel DDPG algorithm.

Throughout the training process of gait optimization, each robot observes the current state from the environment and the Parallel DDPG algorithm calls the state function to obtain the state information of all robots. The actions output by the deterministic policy are executed by all robots separately in the simulated environment. After that, the reward for evaluating the gait is fed back to the Parallel DDPG algorithm. After each round, the experiences in the experience pool are sampled to update the Parallel DDPG model parameters. This process is iterative until termination. The training function algorithm is shown in Algorithm 2.

**Algorithm 2:**
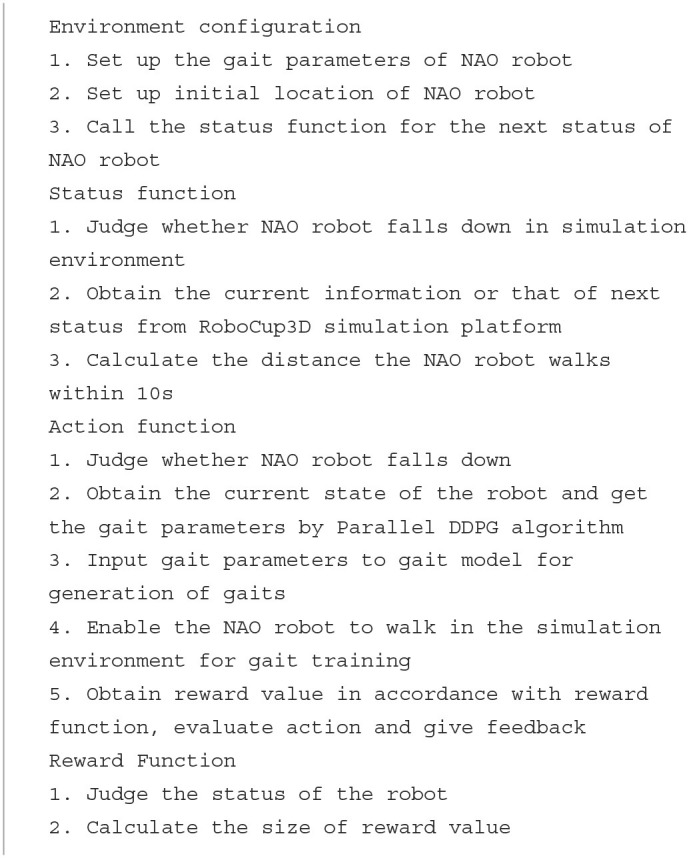
Training algorithm.

### 4.2. Cyclic gait of biped robot

The leg state of biped robot walking is divided into swing phase and support phase. Both phases are a cyclical behavior and switch between the two. A sinusoidal curve is used as a foot height reference for the swing phase. The reference sinusoidal trajectories for the left and right feet are constructed as


(11) $$\begin{array}{l} h_{left}=max\left(0,hsin\left(2\pi t/T+\delta \right)-\Delta h\right), \end{array}$$

(12) $$\begin{array}{l} h_{right}=max\left(0,hsin\left(2\pi t/T+\delta +\pi \right)-\Delta h\right). \end{array}$$

In Equations (11) and (12), changing *h*−Δ*h* can adjust the maximum foot lift height of the biped robot in the swing phase. Δ*h*/*h* is responsible for adjusting the timing of the double support phase. The *T* value is responsible for adjusting the step frequency of biped robot walking. Therefore, different styles of walking gaits of biped robots can be generated using Equations (11) and (12).

**Figure 2:**
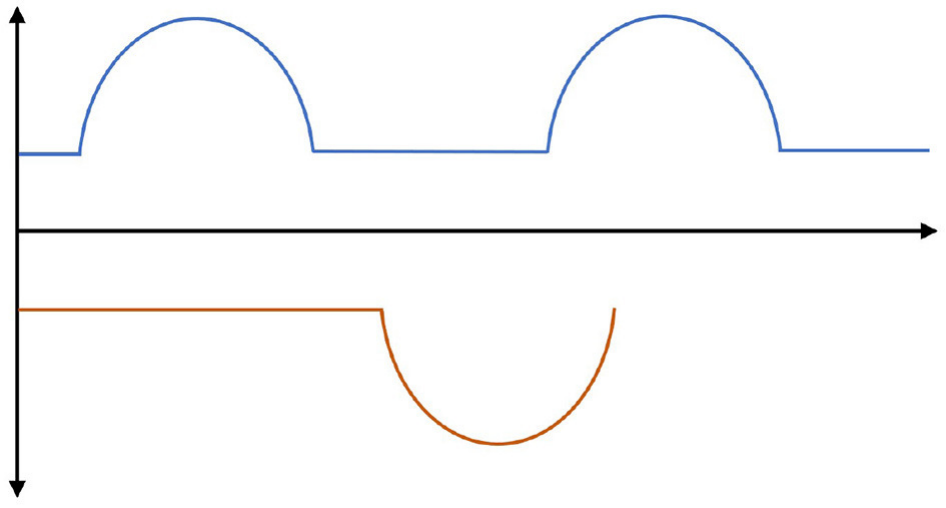
Periodic gait reference trajectories for biped robots. The leg movement of a biped robot during walking is divided into two phases: the swing phase and the support phase. These two phases cyclically alternate with each other. During the swing phase, a sinusoidal curve is used as a reference for the desired height of the foot.

On the basis of Equations (11) and (12), this paper also designs a function punished (ξ_*_) to consider the effects of ground reaction force and foot velocity in cyclic gait, where ξ_*_∈[−1, 1] is the relative progress of the cycle gait.


(13) $$\begin{array}{l} \mathrm{punished}\left(\xi_{*}\right)=\text{a}|\xi_{*}|+\text{b}\frac{d_{\xi_{*}}}{d_{t}}, \end{array}$$

where, *a* and *b* are weighting factors. When the biped robot is in the single support phase, this paper increases the parameter *a* to increase the foot speed and reduce the ground reaction force, which helps to improve the robot's maneuverability. When the bipedal robot is in the dual support phase, boosting parameter *b* can enhance ground reaction force and abate the pace of the two supporting legs, which ultimately serves to optimize robot stability. The specific framework of the periodic gait is shown in Figure 2.

### 4.3. MDP construction

The purpose of MDP construction is to learn a set of strategies to enable biped robots to walk quickly and stably.

1) State space
To set the bipedal robot in motion, the position *q*_*i*_ and velocity $\overset{•}{q_{i}}$ of 8 joints are selected as part of the state space. Considering the kinematics of the biped robot, the following parameters are also added to the state space: the position of the Center of Mass (CoM) of each step, the angular velocity of the torso provided by the torso velocity gyro sensor, the torso acceleration provided by the torso acceleration accelerometer sensor and the calculation of the pressure sensor of both feet, the resulting force is used in the state space. Finally, to meet the requirements of cyclic gait walking for biped robots, the phase vector [*sin*(2π*T*+δ_0_), *cos*(2π*T*+δ_0_)] and velocity command *v*_*x*_ are also taken as part of the state space.
2) Action space
The output of the policy consists of the desired joint positions of the slave joints of the robot (8 per robot). The predictions of the network for the desired joint positions are added to fixed motor biases corresponding to the semi-sitting position of the robot, which are then sent to the lower-level PD controller.
3) Reward
To enable the biped robot to produce a fast and stable periodic gait, a reward function is designed according to the following rules.

(1) Periodic imitation rewards

In order to encourage the robot foot to walk according to the sinusoidal curve, the speed and force of the foot are considered to accelerate the training. The periodic imitation reward function is designed as


(14) $$\begin{array}{l} R_{1}=exp\left(-\frac{1}{0.05^{2}}\overset{2}{\underset{i=1}{\sum }}{\left(h_{ref_{i}}-h_{foot_{i}}\right)}^{2}\right)+r_{gro}+r_{spe}, \end{array}$$

where, *h*_*re*_*f*__*i*__ is the reference height of the left and right feet; *h*_*foo*_*t*__*i*__ is the actual height of the left and right feet; *r*_*gro*_ and *r*_*spe*_ are rewards for foot velocity and force, constructed as


(15) $$\begin{array}{l} r_{gro}=I_{left}^{gro}\cdot F_{left}+I_{right}^{gro}\cdot F_{right}, \end{array}$$

(16) $$\begin{array}{l} r_{spe}=I_{left}^{spe}\cdot S_{left}+I_{right}^{spe}\cdot S_{right}, \end{array}$$

where, *F*_*left*_ and *F*_*right*_ are the ground reaction forces on the left and right feet, respectively; *S*_*left*_ and *S*_*right*_ are the speeds of the left and right feet, respectively.

(2) Stability

In order to maintain the stability of walking, a biped robot needs to keep ZMP inside the support polygon at all times when walking. The support polygon is the smallest polygon area where the biped robot foot is in force contact with the ground. The smaller the distance of ZMP point from the center of support polygon, the more stable the biped robot. A coordinate system is established at the center of support polygon, and the distance between ZMP and the center of support foot is used as a reward.


(17) $$\begin{array}{l} R_{2}=a_{2}*\overset{N}{\underset{k=1}{\sum }}\sqrt{{\left(p_{x}\left(k\right)\right)}^{2}+{\left(p_{y}\left(k\right)\right)}^{2}}, \end{array}$$

where *p*_*x*_(*k*), *p*_*y*_(*k*) are the coordinates of ZMP in each gait sequence, and *a*_2_ is the weight.

(3) Body shaking

In the leg support stage of biped robot, a biped robot is rewarded by criterion that the CoM of biped robot is always at the midpoint of the two-foot coordinate line, so that the torso of biped robot is always stable during the fast walking process.


(18) $$R_{3}=\{\begin{array}{ll} 0 & p_{\text{shake}}<c_{1},\\ c & \text{otherwise}, \end{array}$$

where *c*_1_ is the threshold; *p*_*shake*_ is the deviation between the actual drop point of CoM on X and Y axis and the midpoint of the line connecting centers of feets. The larger the value of *p*_*shake*_, the larger the body sway angle of biped robot during walking, and the more unstable the gait is. In the theoretical case, the best gait results are obtained when the value of *p*_*shake*_ is 0. However, in the actual training process, the coordinate information obtained from RoboCup3D simulation server has some errors, so when *p*_*shake*_ is greater than *c*_1_, the training needs to be punished and the penalty value is the constant *c*.

(4) Speed tracking reward

The velocity tracking reward for biped walking is designed as


(19) $$\begin{array}{l} R_{4}=0.65exp\left(-p_{v}\right)+0.25exp\left(-p_{o}\right), \end{array}$$

where,


(20) $$\begin{array}{l} p_{v}=\frac{||v_{p,x},v_{p,y}-v_{c}|{|}^{2}}{max\left(0.1^{2},0.5||v_{c}|{|}^{2}\right)}, \end{array}$$

(21) $$\begin{array}{l} p_{o}=\frac{sin^{2}\left(0.5<o_{p},o_{u}>\right)}{0.1}. \end{array}$$

Finally, the reward for each step is constructed as


(22) $$\begin{array}{l} R=0.6R_{1}+0.2R_{2}+R_{3}+0.5R_{4}, \end{array}$$

## 5. An optimized algorithm general gait training framework

Recently, various optimization algorithms have emerged one after another. However, different simulation environments and diverse motion models of biped robots have brought challenges to the testing standards of different optimization algorithms. For above-mentioned reasons, an optimized algorithm general gait training framework is designed for various algorithms. The framework defines standard gait optimization tasks and provides a common environment interface for various optimization algorithms. The training framework is mainly composed of RoboCup3D simulation environment, team code and OpenAI Gym.

The optimized algorithm general gait training framework uses RoboCup3D simulation environment to provide simulated physical environments such as stadiums and standard robots. The motion model of UT Austin3D is used as a unified motion model within the framework, and is open to the outside world to adjust parameters of walking engine. The frame is also modularly designed. The algorithm only needs to be connected to framework through the interface of OpenAI Gym to train biped robot gait. When comparing the gait optimization performance of different algorithms, the impact of differences in the simulation environment and motion models is reduced. And there is no need to repeat the development every time, so that researchers can focus more on the optimization of the algorithm.

**Figure 3:**
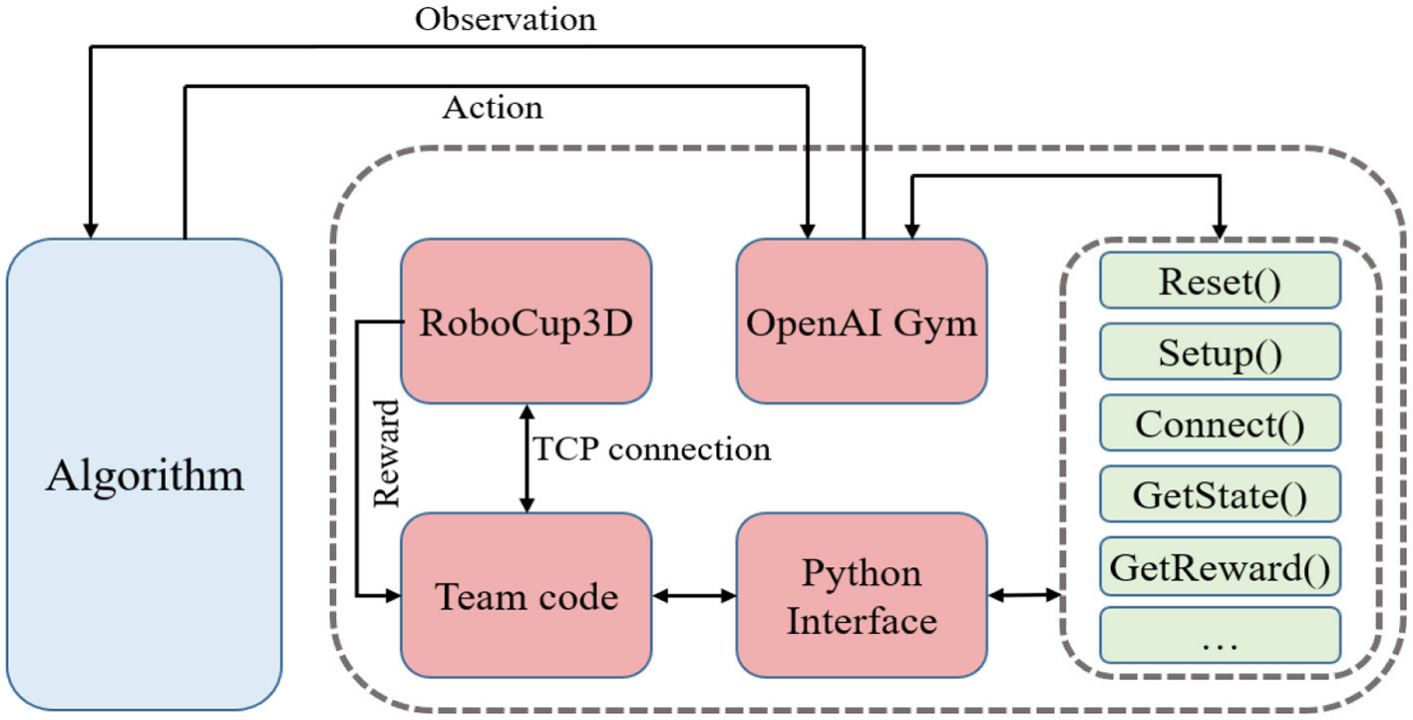
Universal training framework for biped robots. The frame of the biped robot adopts a modular design, allowing for easy replacement of the algorithmic components as needed. To train the biped robot gait, a tested algorithm simply needs to be connected to the framework through the OpenAI Gym interface.

This paper encapsulates functions related to gait and some other functions necessary from the team code into Python interface which are shown in Table 1. Then, according to the abstract definition of environment defined by OpenAI Gym (Torrado et al., 2018), the optimization environment of biped robot is edited and registered locally to the environment collection of Gym, which can provide the function of receiving action, feedback observation and reward for RL algorithm as other existing environments. The system structure of optimization environment for the biped robot is shown in Figure 3. As the team code is built based on the C++, this paper uses ctypes module in Python library to compile C++ code into the form of dynamic linking library, i.e., so file under Linux system.

**Table 1:** Partial list of python interface function.

**Name of function**	**Descriptions of function**
Reset()	Reset the robot to initial state
Step()	Execute the action of walking
Connect()	Build link with simulation server in prior to execution of training
GetState()	Get the current state of the robot
GetReward()	Get award for execution of a pervious action

## 6. Experiment and analysis

**Figure 4:**
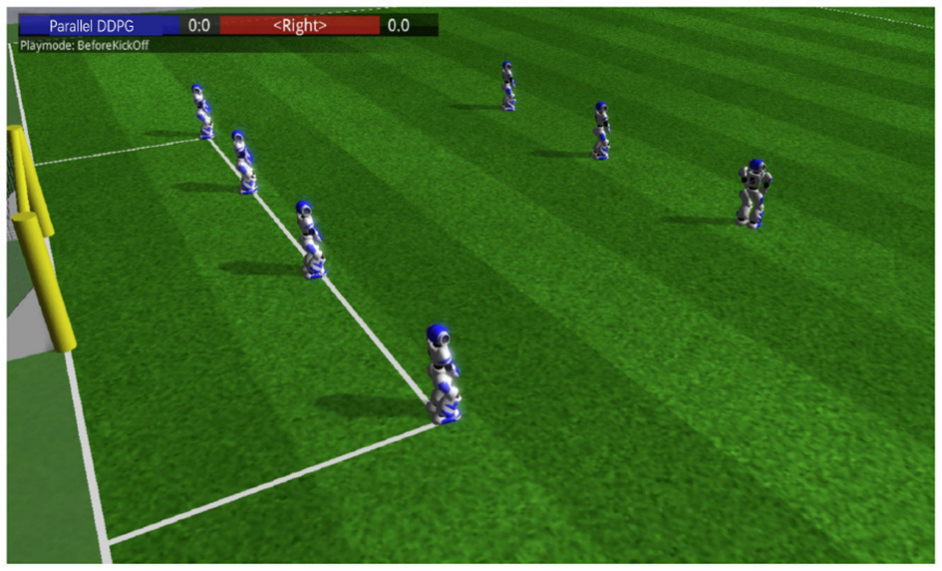
Scene graph of robot gait training. Multiple robots are placed within the training environment, ready for training to begin.

Experiments were conducted under the proposed general gait training framework. In the simulation platform, the range of viewing angles is −120° to 120°. In order to get accurate data during the training process, the setViewCones value of server was changed from 120° to 360°. In the configuration of DDPG network structure, the numbers of input nodes and output nodes were 24 and 27, respectively. Three hidden layers with 300 nodes each were set in this network. The learning rate of actor were 0.0001 and the learning rate of critic were 0.001. The discount factor γ was 0.995 and the τ in the soft update was 0.01. The scene of biped robots using Parallel DDPG algorithm for gait training in RoboCup3D is shown in Figure 4.

To verify the effectiveness of the Parallel DDPG algorithm in gait optimization for bipedal robots, this paper compares the performance of Parallel DDPG, Asynchronous Advantage Actor-Critic (A3C), DDPG, Proximal Policy Optimization (PPO), and CMA-ES algorithms and conducts result analyses for each algorithm.

**Figure 5:**
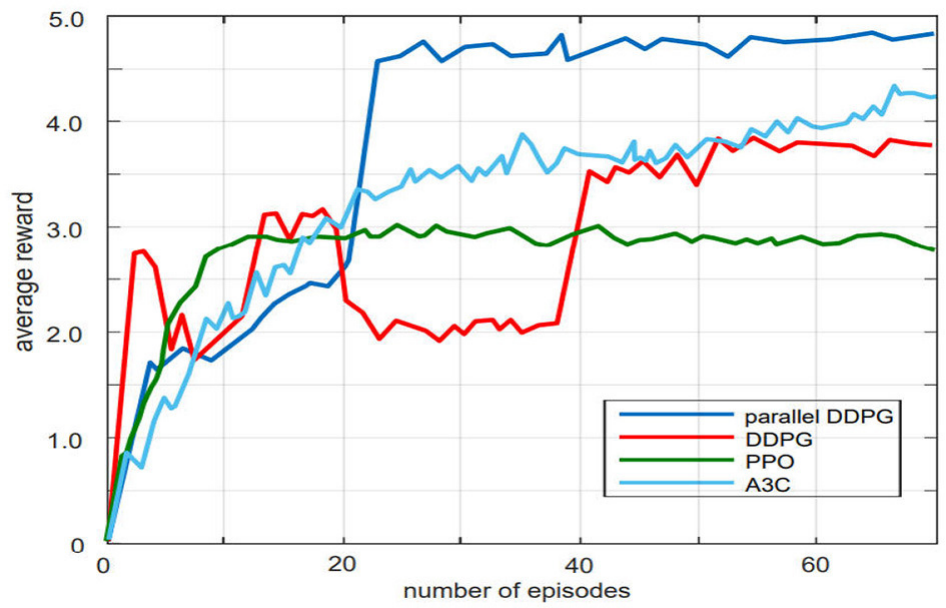
Comparison of parallel DDPG, DDPG, PPO, A3C training curves. The parallel DDPG achieves higher average rewards than the other three algorithms.

Firstly, the learning speed and reward value of the Parallel DDPG, A3C, DDPG, and PPO algorithms were compared. Each algorithm was trained for 21,000 times in the same environment, and the average reward value was recorded every 300 times. The variation of the average reward value during the training process is shown in Figure 5. The Parallel DDPG algorithm achieved the highest average reward value among the four algorithms. In addition, compared to the DDPG and A3C algorithms, the Parallel DDPG algorithm converged to the optimal average reward value the fastest, requiring only 23 episodes. Although the PPO algorithm converged to the optimal average reward in fewer episodes, its average reward value was much lower than that of the parallel DDPG algorithm.

**Figure 6:**
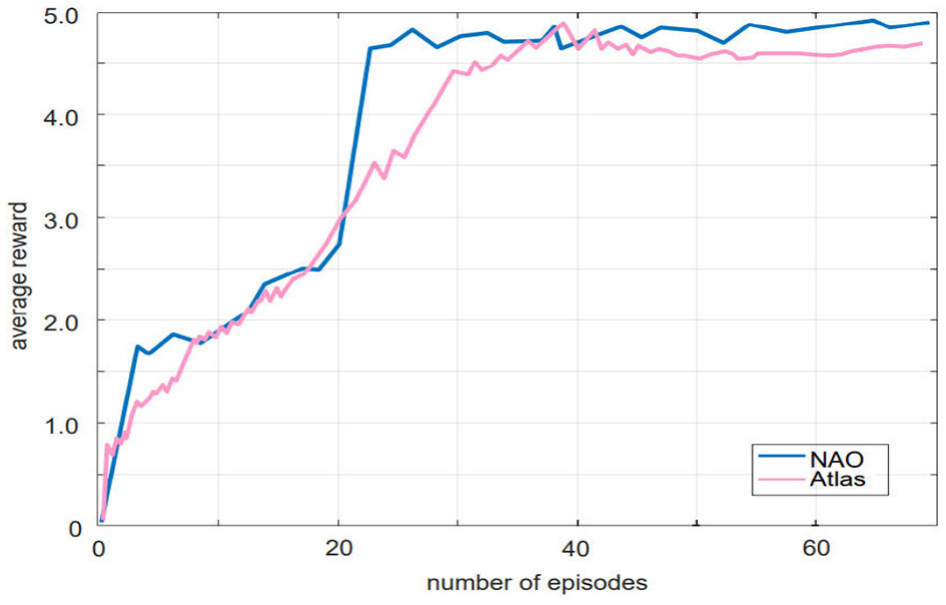
The Parallel DDPG algorithm is utilized to control the training of two biped robots with different physical parameters, resulting in two very similar learning curves.

To evaluate the generalization performance of our proposed algorithm, we conducted additional experiments using another bipedal robot, Atlas, in the Roboschool simulation environment. The simulation environment was different from the soccer field used in our general gait training framework. We trained Atlas using the same 21,000 time steps as in the previous experiment and recorded the average reward values, which were compared to the performance of the NAO robot. As shown in Figure 6, both robots converged under the training of our algorithm, with similar convergence speed and optimal average reward values. These results demonstrate the generalization performance of our algorithm.

As CMA-ES is a global search algorithm that can serve as a benchmark to evaluate performance, and DDPG is the basis for the proposed Parallel DDPG, comparing the two algorithms can demonstrate the effectiveness of the improvement. Therefore, we conducted a more detailed comparison and analysis between the two algorithms.

**Figure 7:**
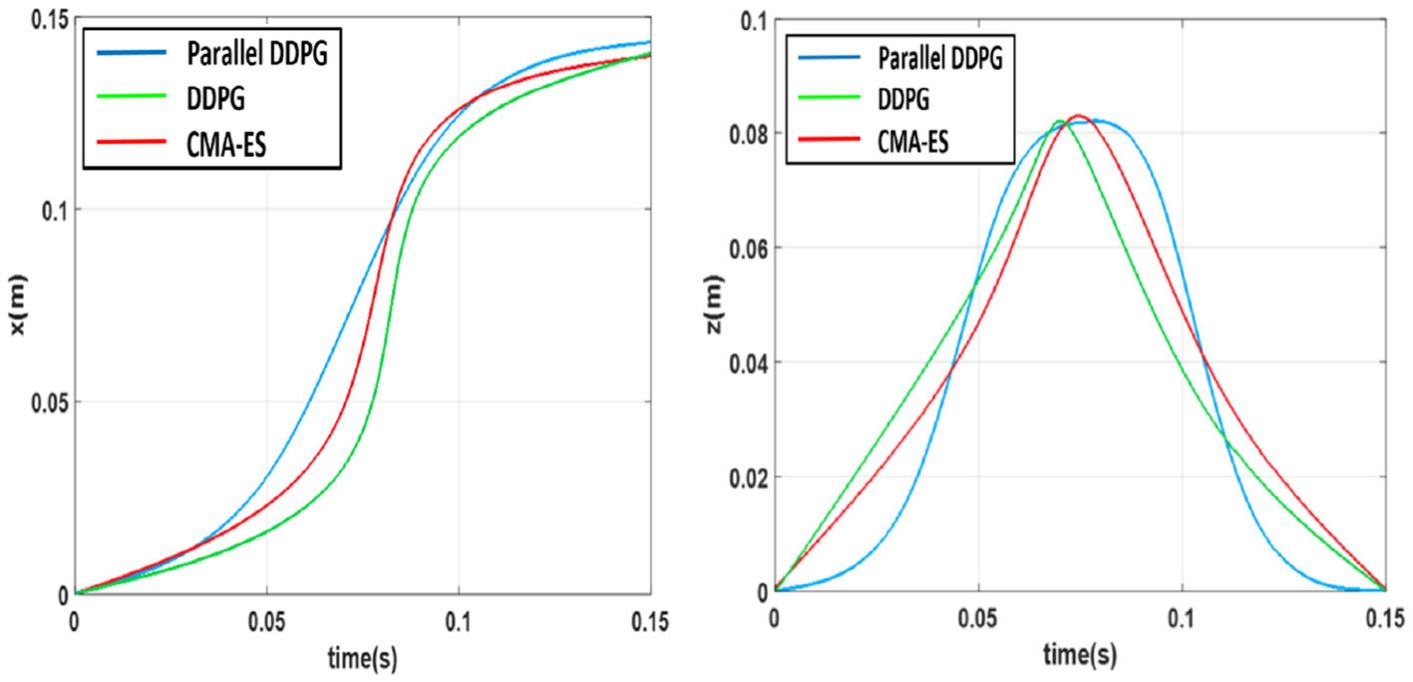
X-axis and Z-axis motion trajectory of swinging leg.

We defined a reference system in which the X-axis points ahead of the robot, the Y-axis points to the left side of the robot, and the Z-axis points above the robot. Using this reference system, we recorded the X-axis and Z-axis trajectories of the walking swinging legs of three algorithms in Figure 7. The Parallel DDPG algorithm optimizes the trajectory of the robot when raising and lowering its feet to ensure stability. As shown in Figure 7, the trajectory obtained by the Parallel DDPG algorithm is smoother than that of the DDPG and CMA-ES algorithms.

**Figure 8:**
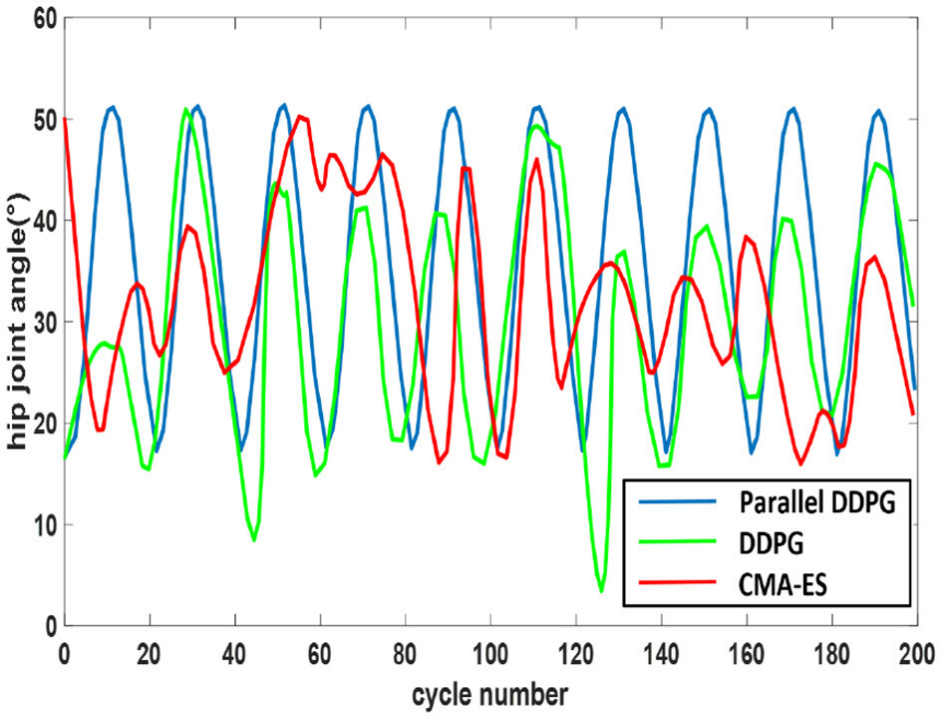
Angle change of hip joint. The trajectory fluctuations of the Parallel DDPG algorithm display the most pronounced periodicity, while those of the other two algorithms exhibit more oscillations.

Figure 8 depicts the alterations in the hip joint angle of the robot following 200 cycles of training (0.05 s per cycle) using the three algorithms. The Figure 8 evidences that the hip joint coordination of the biped robot, utilizing the parallel DDPG algorithm, surpasses that of the other two algorithms, with swift and steady changes.

**Figure 9:**
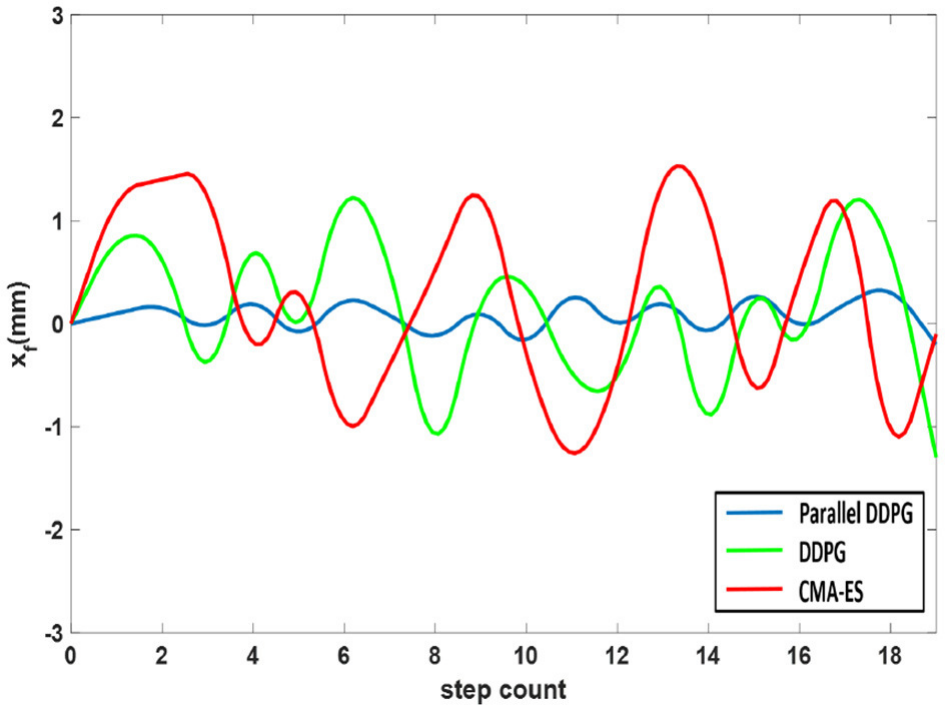
Shake comparison of biped robot trained by three algorithms.

At the onset of the dual support phase, the anticipated coordinates of the CoM are positioned at the midpoint between the two feet. In the initial stage of dual support stage, the vehicle body swing is firstly detected by comparing the center point of two feet and two coordinates of CoM where the equation is *x*_*f*_ = *c*_*x*_−(*x*_*foot*_*R*+*x*_*foot*_*L*)/2. The biped robot utilizing the Parallel DDPG algorithm has less jitter and stable changes, and the results are shown in Figure 9. The Parallel DDPG algorithm displays the narrowest fluctuation range of *x*_*f*_, specifically between −0.3 and 0.4. In contrast, the DDPG algorithm exhibits a fluctuation range of *x*_*f*_ between −1.1 and 1.5, while the CMA-ES algorithm displays a fluctuation range of *x*_*f*_ between −1.3 and 1.7.

**Figure 10:**
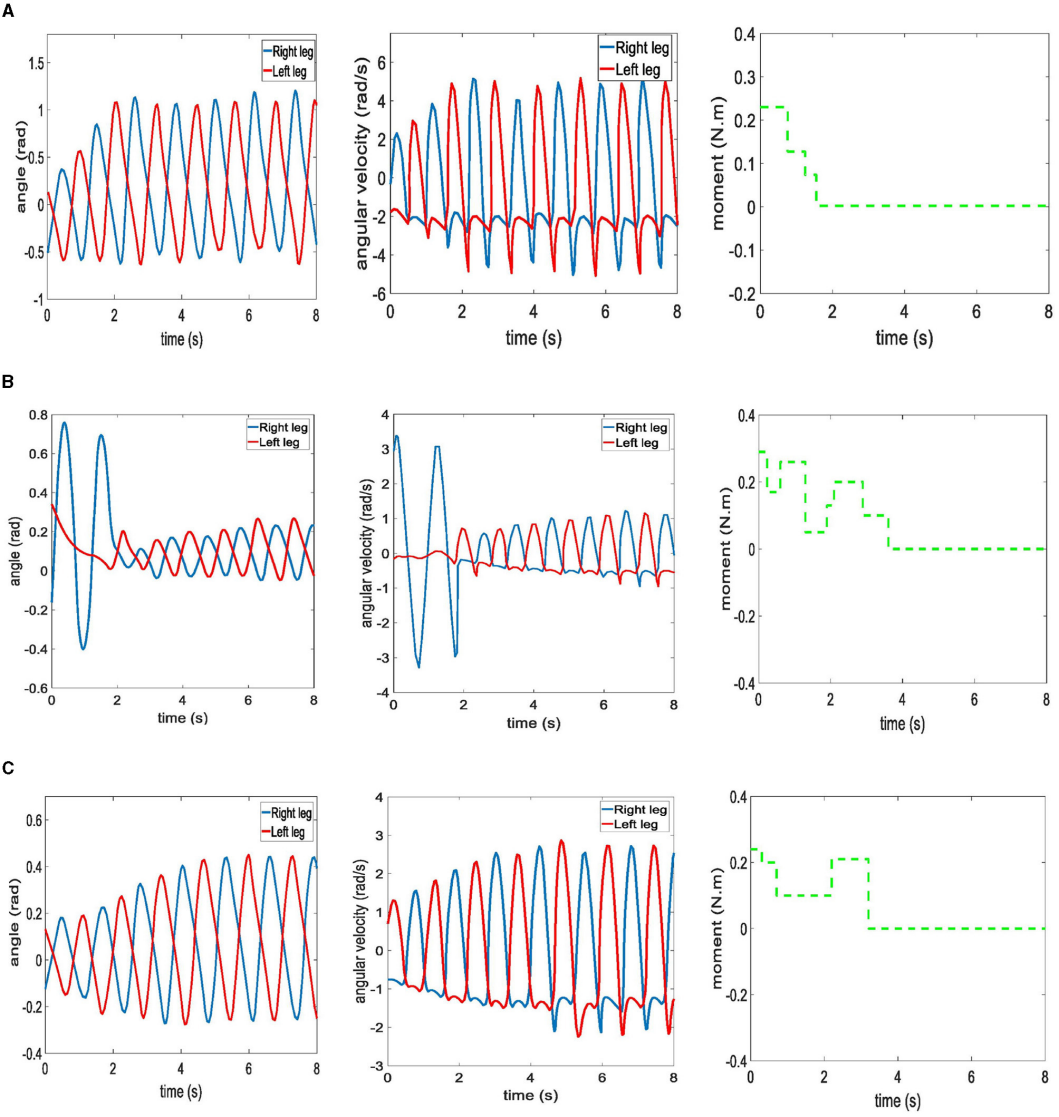
Walking state diagram of biped robot under three algorithms. **(A)** Parallel DDPG. **(B)** CMA-ES. **(C)** DDPG.

The variations of angle and angular velocity between the leg and hip joints when the biped robot walks under the three algorithms is shown in Figure 10. All three algorithms enabled the biped robot to walk stably. The angular velocity of Parallel DDPG algorithm, CMA-ES algorithm, and DDPG algorithm are 2.2, 3.4, and 1.3 rad/s in the first step, respectively. The Parallel DDPG algorithm reaches the maximum angular velocity in the second step, as shown in Figure 10A. The angular velocity of CMA-ES algorithm fluctuates in the early stage and stabilizes after adjustment is shown in Figure 10B. DDPG reaches the maximum value at step 4, as shown in Figure 10C. Moreover, the angular velocity achieved by the Parallel DDPG algorithm surpasses that of the other two algorithms. The aforementioned outcomes indicate that the biped robot trained by the Parallel DDPG algorithm boasts of the longest stride and maintains a rapid walking pace. The biped robot is able to maintain a stable gait when the rate of change of the moment of the swing legs converges. The biped robot using Parallel DDPG algorithm achieved a consistent swing leg moment rate of change in 1.7 s, as shown in Figure 10A. CMA-ES algorithm and DDPG algorithm achieved a consistent swing leg moment rate of change in 3.6 and 3.2 s, respectively, as shown in Figures 10B, C. So Parallel DDPG algorithm made the biped robot reach the stable walking state in the shortest time.

**Figure 11:**
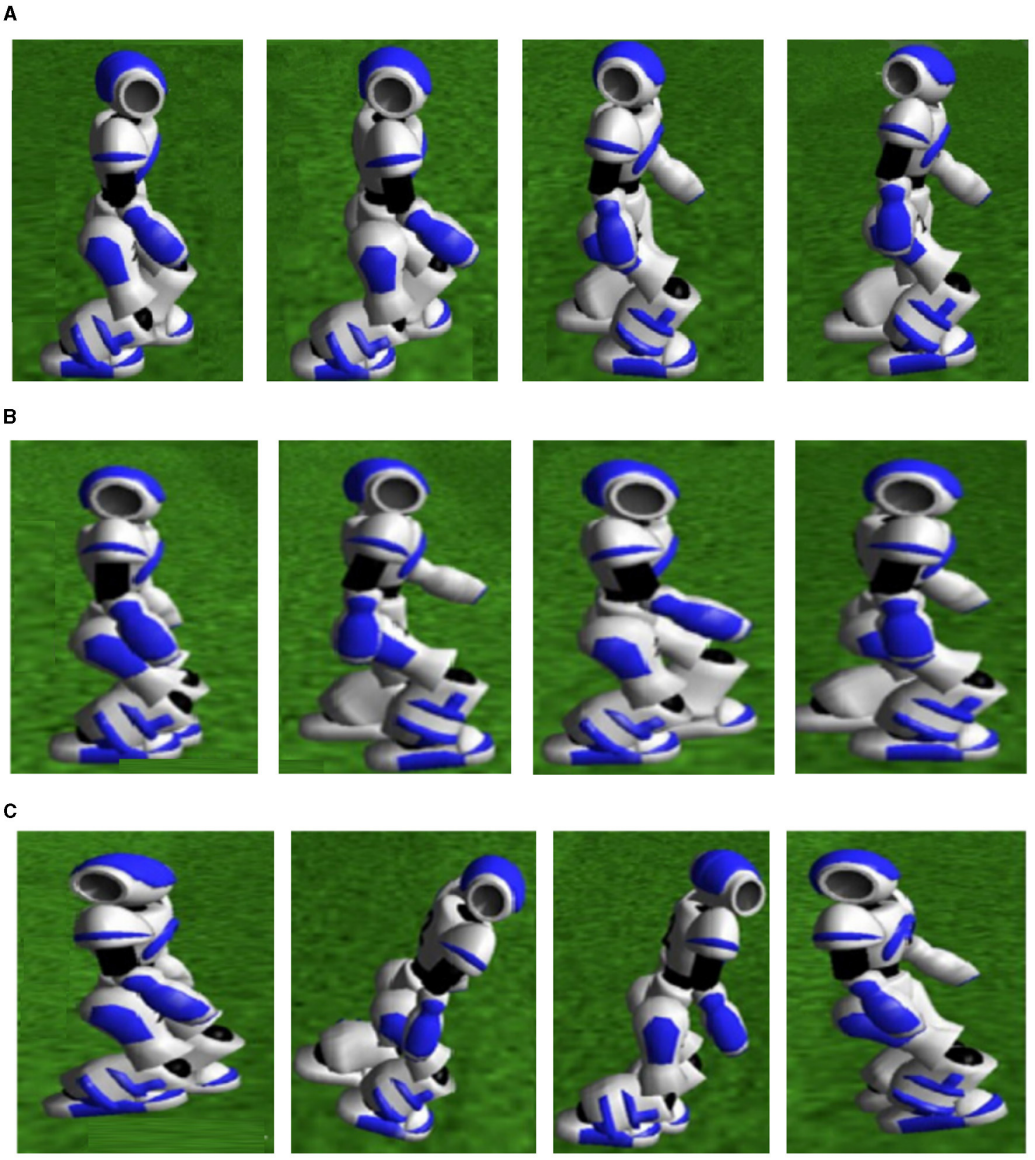
Biped robots walking details of three algorithms. **(A)** Parallel DDPG. From left to right: t = 6.12 s, t = 6.25 s, t = 6.37 s, t = 6.50 s. **(B)** DDPG. From left to right: t = 6.12 s, t = 6.25 s, t = 6.37 s, t = 6.50 s. **(C)** CMA-ES. From left to right: t = 6.12 s, t = 6.25 s, t = 6.37 s, t = 6.50 s.

Figure 11 shows the walking details of three algorithms tested on the RoboCup3D simulation platform for training a bipedal robot. The time point selected is in the middle and late stages of training, which is a period suitable for comparison. The CMA-ES algorithm resulted in a very unstable state, as shown in Figure 11C, with the bipedal robot's body wobbling. In contrast, the Parallel DDPG and DDPG algorithms could maintain stability of the bipedal robot's torso due to autonomous learning based on UT Austin3D. The Parallel DDPG algorithm obviously showed a more stable pose.

**Figure 12:**
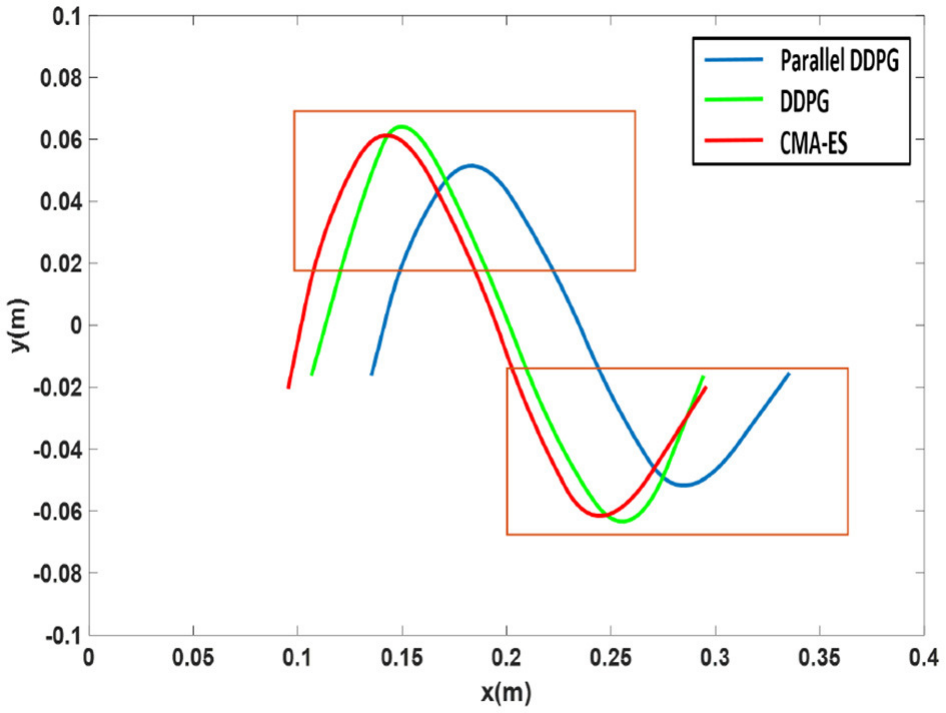
ZMP trajectories optimized by three algorithms.

When using three algorithms to walk, ZMP trajectories of biped robot are shown in Figure 12. ZMP trajectories of DDPG and CMA-ES algorithms are close to the edges of support polygons. However, the ZMP trajectory profile of the Parallel DDPG shifts between the support polygons, which results in a large ZMP stability margin for the bipedal robot.

**Figure 13:**
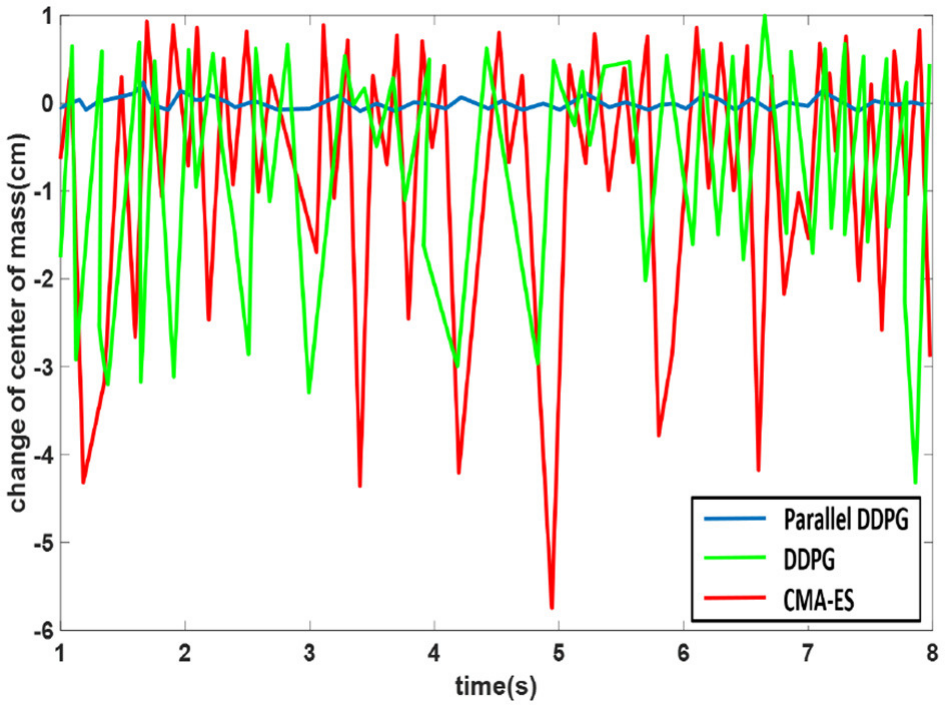
The changes in the CoM landing points for three algorithms.

Figure 13 portrays the CoM landing point variations for the three methods. Throughout the biped support phase, the CoM utilizing the Parallel DDPG algorithm remains consistently stable at the center of the connection between the two legs, whereas the CoM using DDPG and CMA-ES displays instability.

Three algorithms were tested when the biped robot walked 15 meters away. Table 2 presents the average outcomes of 100 tests, indicating that a biped robot incorporating the Parallel DDPG algorithm requires less time and exhibits a faster walking speed for the identical distance. The average speed of the biped robot utilizing the Parallel DDPG algorithm reached 0.85 m/s, whereas the average speed of DDPG algorithm was 0.75 m/s, and the average speed of CMA-ES algorithm was 0.71 m/s.

**Table 2:** Straight speed comparison.

**Algorithm**	**Distance (m)**	**Time (s)**			**Speed (m/s)**		
		**Average**	**Max**	**Min**	**Average**	**Max**	**Min**
Parallel DDPG	15	17.63	18.99	17.05	0.85	0.88	0.79
DDPG	15	20.07	22.38	18.75	0.75	0.80	0.67
CMA-ES	15	24.03	26.79	23.08	0.62	0.65	0.56

**Figure 14:**
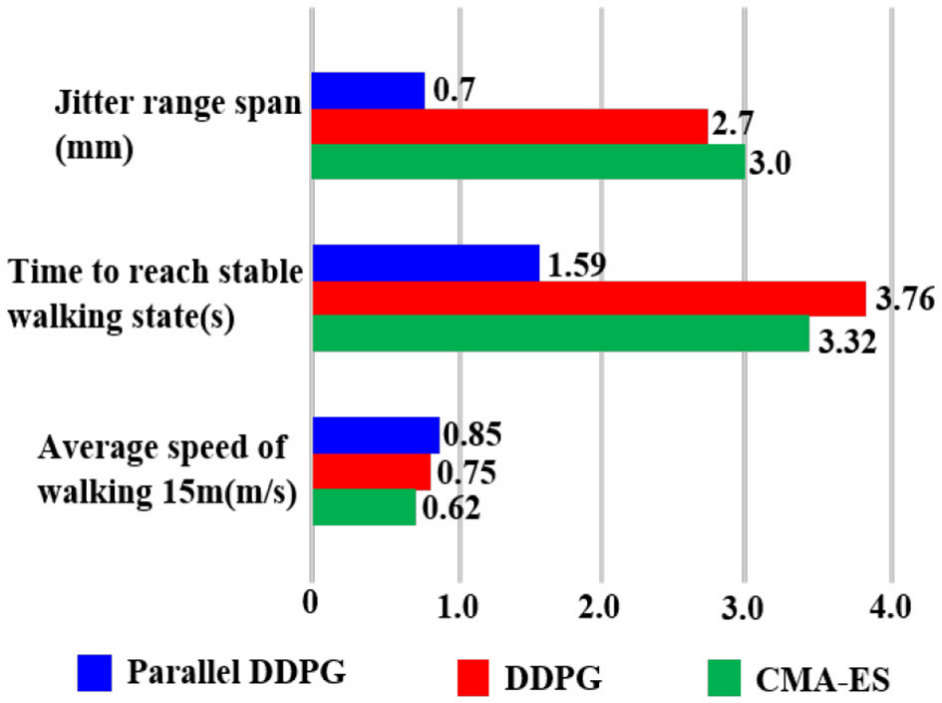
This figure describes the performance comparison of the three algorithms when controlling the biped robot to walk.

Finally, a summary of the performance of the three algorithms that were further analyzed is shown in Figure 14. Figure 14 summarizes the performance of the three algorithms in terms of jitter range span, average speed of walking 15 m, and time to reach stable walking state. The results in the figure align well with our previous summary of the performance of the three algorithms.

## 7. Conclusion

In this paper, a parallel heterogeneous DRL algorithm is proposed to optimize the walking gait of a biped robot. The parallel training method improves the training speed. The heterogeneous experience replay is used to replace the traditional experience replay mechanism to increase the efficiency of experience application. Sinusoidal curves have been used as a reference for the foot of the bipedal robot, which increases the potential of excellent empirical sample generation. The proposed training framework for biped robots gait optimization provides a unified test environment for gait training of different algorithms and reduces the effects of simulation environment and motion model differences. Experiments demonstrate that the Parallel DDPG algorithm enables a faster and more stable gait. However, it is important to note that there are limitations to this study due to the gap between simulation and practical application. In future research, we plan to extend the proposed method to a real biped robot and investigate various styles of periodic gaits. Overall, our results provide valuable insights into the development of gait optimization algorithms for biped robots, and we believe that our approach can be further improved and refined in future research.

## Data availability statement

The raw data supporting the conclusions of this article will be made available by the authors, without undue reservation.

## Author contributions

CL and ML contributed to methodology and programming of the study. ML wrote the first draft of the manuscript. CT wrote sections of the manuscript. All authors contributed to manuscript revision, read, and approved the submitted version.
